# Immobilizing partial denitrification biomass and redox mediators to integrate with the anammox process for nitrogen removal†

**DOI:** 10.1039/c9ra05525h

**Published:** 2019-12-13

**Authors:** Chuan He, Li'e Wei, Faying Lai, Chunhuo Zhou, Guorong Ni, Jianmin Hu, Xin Yin

**Affiliations:** Nanchang Key Laboratory of Nutrition Management of Crops, Prevention and Controlling of Agricultural Non-point Source Pollution, College of Land Resource and Environment, Jiangxi Agricultural University Nanchang 330045 PR China yinxin1081@163.com; Jiangxi Provincial Key Laboratory of Water Resources and Environment of Poyang Lake, Jiangxi Institute of Water Sciences Nanchang 330029 PR China

## Abstract

In this study, immobilizing partial denitrification biomass and redox mediators to integrate with the anammox process for nitrogen removal was investigated. Three redox mediators (RMs), namely, 2-methyl-1,4-naphthoquinone (ME), anthraquinone (AQ) and 1-dichloroanthraquinone (1-AQ) were catalyzed to reduce nitrate to only nitrite by denitrification to integrate with the anammox process for nitrogen removal. First, our experimental results showed that there were 35.8, 42.2 and 53.0 mg-N L^−1^ nitrite accumulation values with the addition of ME, AQ and 1-AQ, respectively, at the dose of 75 µM by the denitrification process at C/N = 2, which were 25.6%, 48.2% and 86.1% higher than that of the control without the addition of any RMs. Nitrate reductase activities were higher than that of nitrite reductase affected by RMs, which was the main reason for nitrite accumulation and further maintenance of the anammox process. Second, owing to the stable nitrite production by the partial denitrifying biomass with the addition of 1-AQ, the nitrogen removal rate of the reactor that integrated the partial denitrification and anammox process reached 1788.36 g-N m^−3^ d^−1^ only using ammonia and nitrate as the influent nitrogen resource in the long-term operation. Third, the 16S rDNA sequencing results demonstrated that *Yersinia frederiksenii* and *Thauera* were the primary groups of the denitrifying biomass, which were considered the dominant partial denitrification species.

## Abstract 2

In this study, immobilizing partial denitrification biomass and redox mediators to integrate with the anammox process for nitrogen removal was investigated.

## Graphical abstract

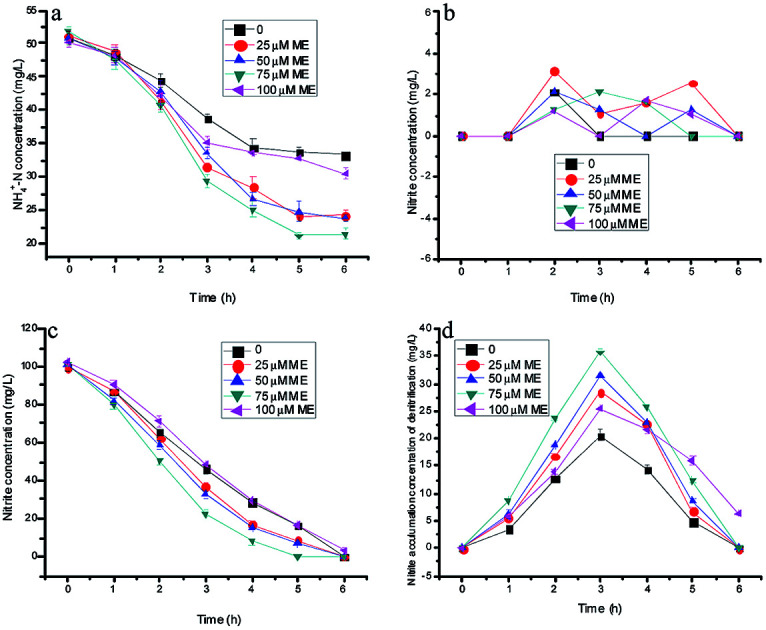

## 1. Introduction

The use of anaerobic ammonium oxidation (anammox) bacteria is a potential biological nitrogen removal technology, which employs nitrite and ammonium as the electron acceptor and donor.^1,2^ Owing to the low organic carbon requirements and little sludge production, the anammox process has attracted much attention for its significant advantages compared to the traditional nitrification/denitrification nitrogen removal process, and it is widely studied for its promising application as an alternate process to the nitrogen removal processes in recent years.^3–6^

Partial nitrification has been widely studied for an application as an anammox pretreatment technology. Due to the inhibition of free nitrous acid, high oxygen, high pH, free ammonia, high temperature and alkali on nitrite oxidizing bacteria, many studies have been reported for controlling the partial nitrification process.^7–9^ Zeng *et al.* realized that nitrite could accumulate if the DO concentration was controlled at 0.3–0.7 mg L^−1^.^10^ Also, mainstream partial nitrification/anammox was proposed as pivotal for a more sustainable treatment of municipal wastewater.^11,12^ Hoekstra *et al.* demonstrated that the total nitrogen removal rate was 0.223 ± 0.029 kg N (m^3^ d)^−1^ during stable process operations at summer temperatures (23.2 ± 1.3 °C).^11^ However, unsatisfactory accumulation could occur from the partial nitrification process on account of the easy recovery of the nitrite-oxidizing bacteria from oxygen limitation when DO was increased during continuous aeration. In addition, Zeng *et al.* realized that a low DO concentration could bring down the activities of the aerobic ammonia-oxidizing bacteria, resulting in low nitrite accumulation by the partial nitrification process.^13^ Evidently, it was not easy to obtain stable nitrite accumulation and control the further oxidation to nitrate by partial nitrification.

Actually, the accumulation of nitrite during the denitrification process has been demonstrated frequently and drawn much attention in recent years, which can offer another way to supply an electron donor for anammox. Kalyuzhnyi *et al.* reported the anammox process with autotrophic denitrifying conditions using sulphide as an electron donor for the production of nitrite from nitrate within an anaerobic biofilm.^14^ Recently, a high nitrite accumulation *via* denitrification sludge was cultivated and maintained in a long-term operation.^15–17^ Cao *et al.* demonstrated that nitrite could be produced in a partial denitrifying upflow sludge bed reactor equipped with gas automatic circulation.^15^ The peak value of the nitrite accumulation reached 102.6 mg L^−1^. However, there were so many studies showing that the nitrite production rate was relatively low with the partial denitrification application, and it could not meet the requirement of anammox for nitrogen removal.^18,19^ Therefore, a more robust nitrite production with partial denitrification should be developed.

Recently, the effects of redox mediators (RMs) were studied on the anaerobic degradation of inorganic and organic contaminants.^20,21^ Also, many options focused on the effects of RMs on nitrogen removal by the denitrification process. Aranda-Tamaura *et al.* proved that 1-dichloroanthraquinone (1-AQ) could accelerate the nitrate reduction rate by sulfur autotrophic denitrification.^22^ Guo *et al.* demonstrated that anthraquinone (AQ) immobilized by calcium alginate could catalyze the denitrification process. It was well known that the nitrogen removal performance of denitrification bacteria would show different tendencies with different kinds of RM addition.^23^ The addition of RMs might expedite the electron transfer between the electron acceptors and donors. Kelso *et al.* found that the competition between nitrate reductase (NR) and nitrite reductase (NIR) would result in the accumulation of nitrite during the denitrification process.^24^ Furthermore, our previous study proved that the unbalanced NR and NIR activities affected by RMs were considered the main reason for nitrite accumulation, and further lead to different nitrogen removal performances with different RM additions.^25^ Hence, the discrepant effects of RMs on different enzymes might lead to different substrate converting rates, and the accumulation of nitrite accumulation by the denitrification process. Thus, partial denitrification controlling by redox mediators integrating the anammox process for nitrogen removal was possible.

The main objective in this study was to investigate the feasibility of nitrite accumulation by partial denitrification with RM addition. Moreover, the mediated partial denitrification with RMs by immobilizing technology would couple with the anammox process for nitrogen removal.

## 2. Materials and methods

### 2.1 Inoculum and substrate medium

Seed denitrification sludge was taken from a full-scale municipal wastewater treatment plant (Qingshanhu Wastewater Treatment Plant, Nanchang, China). The sludge was cultivated using glucose as the only electron donor in a lab-scale, up-flow anaerobic sludge blanket (UASB) reactor (2.5 L) year round. The residual nitrogen was mainly in the form of nitrite at a concentration of about 20 mg L^−1^. During the cultivation period, NO_3_^−^–N was supplied in the form of KNO_3_. The substrate medium was described by Yin *et al.*^25^ The anammox biomass used for experiments was obtained from a laboratory-scale anammox upflow column reactor. The anammox bacteria of KSU-1 strain (AB057453.1) accounted for about 70–75% of the total biomass in seed biomass by FISH observation. The composition of the trace mineral medium has been previously described by Kang *et al.*^26^ The incubation medium consisted of trace mineral elements, KHCO_3_ acting as the only carbon source, ammonium in the form of (NH_4_)_2_SO_4_ and nitrite supplied by NaNO_2_. All regents were purchased from the Sinopharm group chemical reagent co., LTD.

### 2.2 Analytical methods

Water samples were filtered through filters for the determination of the NH_4_^+^–N, NO_2_^−^–N and NO_3_^−^–N concentrations. The NO_2_^−^–N and NO_3_^−^–N concentrations were measured by ion-exchange chromatography (ICS-900, DIONEX, USA). The mixed liquor volatile suspended solid (MLVSS) and mixed liquor suspended solid (MLSS) concentrations were measured by standard methods.^27^ pH measurements were performed by a digital pH meter (PHS-25, Leici Company, China), while DO was measured by a digital DO meter (YSI, Model 55, USA).

### 2.3 Measurement of NR and NIR activity

2 g (wet weight) denitrification biomass was obtained from the reactor for the crude enzyme activity measurement. The crude enzyme extraction procedure was achieved as described by Qiao *et al.*^28^ The protein concentration was measured using bovine serum albumin as a standard described by Bradford.^29^ The NR and NIR activities were measured by the methods according to Kataoka *et al.*^30^ and Yin *et al.*^31^ The reducing rate of nitrate and nitrite was characterized by the NR and NIR activities. The NR and NIR enzyme activities were defined as the reduction of 1 µmol of nitrate and nitrite per minute.

### 2.4 Batch experiments

Three representative redox mediators, including 2-methyl-1,4-naphthoquinone (ME), AQ and 1-AQ, were used. All kinds of RMs had one or more six-membered cyclic diketones with two carbonyl groups containing two double bonds. Fig. S1† illustrates the structure of the three RMs. To avoid the toxicity of RMs on the anammox biomass reported by Qiao *et al.*,^28^ the RMs and denitrification biomass were immobilized in entrapping beads. The immobilization method was described by Qiao *et al.*^28^ Batch experiments were carried out in 120 mL serum bottles loaded with 100 mL substrate medium. To remove the redundant nitrogen element, anammox biomass obtained from the UASB reactor was washed three times with mineral medium. Then, both entrapping beads and anammox sludge were transferred to the serum bottles. In addition, single denitrification batch experiments were only put into entrapping beads in the serum bottles to explore the nitrite accumulation of the denitrifying biomass. The temperature was maintained at 37 ± 1 °C, while the initial pH was adjusted to 7.5. The serum bottles were blown with nitrogen gas to achieve anaerobic conditions. The initial ammonia and nitrate concentrations were set at about 50 and 100 mg-N L^−1^, and the reaction time lasted 6 h in each integrating test. However, the initial nitrate concentration was set at 100 mg-N L^−1^ in the single denitrification batch experiments. Another substrate medium used in the batch experiments was the same as that of the lab-scale UASB reactor. Glucose and KHCO_3_ were the carbon sources for denitrification and anammox biomass. Water samples were taken every 2 h and immediately stored in a refrigerator at 4 °C for analysis. All batch tests were carried out in triplicate.

### 2.5 Continuous experiments

**Fig. 1:**
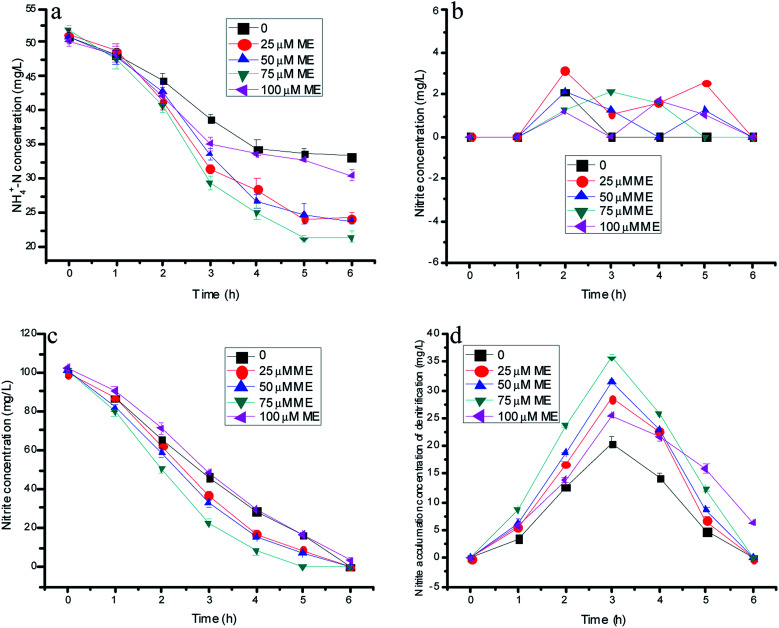
The variation of nitrogen concentration with different doses of ME addition in batch experiments.

The working volumes were about 0.3 L with a height of 15 cm and an inner diameter of 5 cm. Four reactors contained 15 g (wet weight) anammox and 30 g entrapping bead (immobilization denitrification biomass and appropriate RMs). At the beginning of the reactor start-up, the influent NH_4_^+^–N and NO_3_^−^–N doses were 50 mg L^−1^ and 100 mg L^−1^. The initial hydraulic retention time (HRT) was 6 h, corresponding to the nitrogen-loading rate (NLR) at 600 g-N m^−3^ d^−1^. Four reactors were continuously fed with the same media, and the influent was purged with 99.5% N_2_ to maintain dissolved oxygen (DO) below 0.5 mg L^−1^. The influent pH was adjusted to 7.0 ± 0.2 by dosing 2 M HCl, and the temperature was maintained at 35 ± 1 °C using a water bath. Fig. 1 shows the schematic diagram of the continuous experiments.

### 2.6 DNA extraction, amplification of 16S rRNA genes, cloning and sequencing

Total DNA was extracted from enriched culture samples (approximately 0.3 g) with a PowerSoil® DNA Isolation Kit (Mobio, USA), as described previously.^32,33^ The 16S rRNA gene was amplified using the universal forward primer 27F (5′-AGAGTTTGATCMTGGCTCAG-3′) and reverse primer 1492R (5′-TACGGYTACCTTGTTACGACTT-3′).^34^ The PCR procedure was as follows: initial denaturation at 94 °C for 5 min and 35 cycles consisting of denaturation at 94 °C for 1 min, primer annealing at 53 °C for 1 min and extension at 72 °C for 90 s, followed by a final elongation step of 72 °C for 7 min. The PCR products were analyzed on a 1% (w/v) agarose gel and purified using an AxyPrep™ DNA Gel Extraction kit (Axygen Biosciences, Hangzhou, China). PCR products were ligated into a pMD 18-T vector (TaKaRa Bio Inc., Dalian, China), and transformed into *Escherichia coli* DH5α cells. Plasmid insert-positive recombinants were selected using 5-bromo-4-chloro-3-indolyl-β-d-galactopyranoside (X-Gal) and isopropyl-β-d-thiogalactopyranoside (IPTG) LB indicator plates with 100 µg mL^−1^ ampicillin. The primer RV-M was used to sequence plasmid inserts by BGI.

The nucleotide sequences were compared to those from the GenBank using a BLAST search of the National Center for the Biotechnology Information server. The sequences in this study and reference sequences retrieved from GenBank were aligned with the CLUSTAL_X program. Phylogenetic trees were confirmed by further analysis using MEGA software version 5.0 with the neighbor-joining (NJ) method.

## 3. Results and discussion

### 3.1 Effects of ME on the nitrogen removal rate

Fig. 1a–c showed nitrogen variations with time by the immobilizing denitrification biomass and various ME dosing concentrations integrated with the anammox process. The effects of ME on the nitrite accumulation by the single denitrification process are depicted through Fig. 1d. As shown in Fig. 1a, the ammonia concentration of control decreased from 50.8 to 33.3 mg L^−1^ in the absence of influent nitrite. In the experiments, the nitrite resource only could come from nitrate reduction by denitrification biomass, which was similar to our previous research. However, it was regrettable that the nitrite accumulation by normal denitrification was too little for the anammox process. Because of the underproduced nitrite product in control tests, the ammonium concentration consumption by anammox decreased within the first 4 h of reaction, but still stabilized after another 2 hours. Additionally, NO_3_^−^–N presented a declining trend with almost complete consumption within 6 and 5 h, respectively (Fig. 1c). Remarkably, the nitrate concentration decreased faster from 100 to 8.78, 6.65 and 0 mg L^−1^ at the ME dosing concentrations of 25, 50 and 75 µM compared with the control of 16.4 mg L^−1^ within 5 h reaction time. However, in comparison with the control at 20.5 mg L^−1^, the peak value of nitrite accumulation reached 28.5, 31.5 and 35.8 mg L^−1^ at the ME dosing concentrations of 25, 50 and 75 µM, as shown in Fig. 1d. Thus, the maximum ammonia removal efficiency by anammox was 58.5% with 75 µM ME addition benefitting from much more nitrite accumulation of denitrification, which was 69.8% higher than that of the control (34.5%). However, the ammonia removal efficiency of the anammox bacteria showed a decreasing tendency when the ME concentration increased to 100 µM, causing the decline of nitrite production by denitrification. Evidently, the ammonia removal efficiency of anammox was related to the nitrite accumulation of denitrification, which was affected by ME. The nitrate consumption and nitrite production of the denitrification biomass with 100 µM ME addition may be due to the original toxicity of the RMs to bacteria with a high level dose. There was low nitrite accumulation on the batch experiments existing in both anammox and the denitrification biomass with different doses of ME addition, for the reason that there might be not enough nitrite production supplying anammox and the denitrification reaction at the same time (Fig. 1b).

### 3.2 Effects of AQ on the nitrogen removal rate

**Fig. 2:**
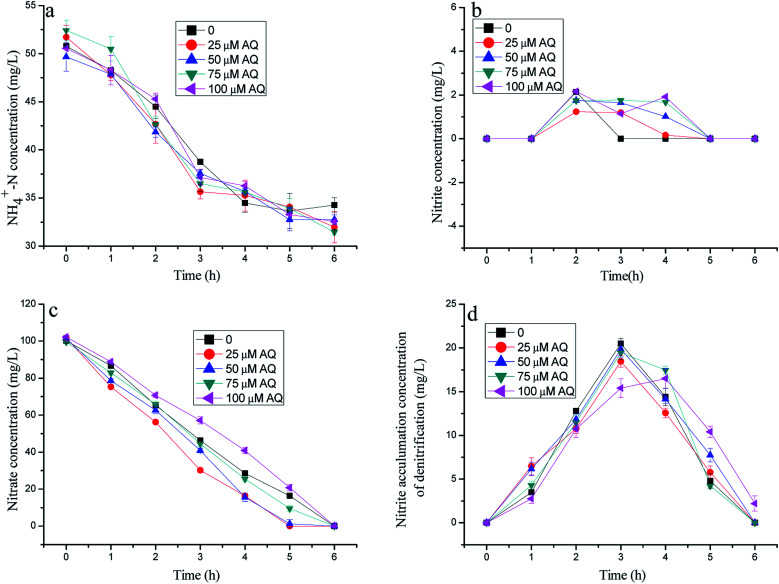
The variation of nitrogen concentration with different doses of AQ addition in batch experiments.

As described in Fig. 2, the nitrogen removal results with different AQ dose additions practically showed similar nitrate consumptions, but different ammonia removal patterns. Fig. 2c presents the residual nitrate at 0, 1.32, 9.42, 20.8 mg L^−1^ with 25, 50, 75, 100 µM AQ addition, respectively, corresponding to 16.4 mg L^−1^ NO_3_^−^–N of the control remaining after 5 h reaction time. These results demonstrated that an appropriate AQ addition could enhance the nitrate consumption by the denitrification biomass. Nonetheless, it appeared to have almost exactly the same ammonia removal performance, meaning the identical anammox activities with different AQ dose additions, including that of the control (Fig. 1a). The AQ addition did not lead to more nitrite accumulation of the denitrification process as shown in Fig. 1d, which might be the main reason for the stagnation of the anammox reaction on account of insufficient substrates. The exact reason for different nitrite accumulation performances of the denitrification reaction at different kinds of RMs might be in connection with the effect of RMs on the key enzymes, such as the NR or NIR enzymes (discussion later). In addition, the low nitrite concentration detection in the tests with mixed bacteria indicating the nitrite production by denitrification was hardly lack for anammox biomass within the reaction time, as shown in Fig. 1b.

### 3.3 Effects of 1-AQ on the nitrogen removal rate

**Fig. 3:**
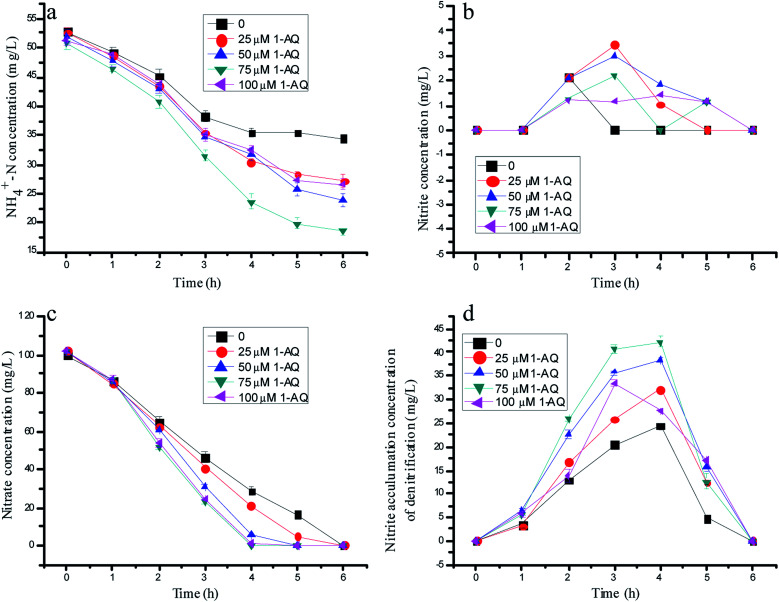
The variation of nitrogen concentration with different doses of 1-AQ addition in batch experiments.

Fig. 3 depicts the effects of 1-AQ on the nitrogen removal performance of batch experiments. When the initial 1-AQ addition increased from 0 to 75 µM, the rest of the ammonia concentration of the batch tests decreased from 34.5 to 18.5 mg L^−1^ after 6 h of reaction time (Fig. 1a). Conspicuously, it was found that the supreme ammonia removal efficiency reached 63.6% with 75 µM 1-AQ addition, which was 83.80% higher than that of the control group. Identically, the higher RM dose addition (100 µM) could inhibit the ammonia removal performance by anammox bacteria resulting from the least nitrite accumulation by denitrification biomass. As illustrated in Fig. 3c, the nitrate concentration decreased profoundly in general, as with AQ particularly at a dosing 1-AQ concentration beyond 50 µM. Nitrate at a 1-AQ concentration of 50, 75 and 100 µM was almost removed completely, but remained 28.5 and 20.8 mg L^−1^ at a concentration of 0 and 25 µM after 4 h reaction time. Obviously, the RM addition with a certain concentration range accelerated the nitrate conversion by the denitrification biomass. Importantly, the nitrite production could be improved at a certain 1-AQ concentration due to the sharp nitrate consumption, as described in Fig. 3d. The peak nitrite accumulation reached 42.18 mg L^−1^ with a 75 µM 1-AQ addition, which was 1.73-fold as much as that of the control in the denitrification experiments. Thus, owing to the much greater nitrite amount resulting from denitrification, the higher ammonia removal by the anammox reaction would appear. In our study, for highlighting the effects of RMs, the NO_3_^−^–N concentration of the influent only was 100 mg L^−1^, so that a particularly high nitrite accumulation could not appear, similar to the study of Cao *et al.*^15^

Our preliminary research made a point that RMs could really affect the activities of key enzymes resulting in the dissimilarity of the NR and NIR activity, which might be the main reason for nitrite accumulation during the denitrification process.^31^ The effects of 1-AQ on the denitrification biomass and key enzyme activities could support the viewpoint again. The NR activity of 64.1 µM-N mg protein^−1^·min^−1^ before RM addition was a little different from the NIR activity of 52.26 µM-N mg protein per min. The RM addition enlarged the gap of the NR and NIR activities. With 75 µM 1-AQ addition, the NR (108.46 µM-N mg protein per min) and NIR (71.46 µM-N mg protein per min) activities were enhanced 69.1% and 36.7% higher than those of the control, as depicted in Fig. S2.† Undoubtedly, nitrite can be produced excessively by partial denitrification, owing to the discrepancy between the enhanced NR and NIR activities with a certain 1-AQ dose addition, and might be used as an electron acceptor stabilized by anammox bacteria to oxidize ammonium.

### 3.4 Effects of the C/N ratio on the nitrogen removal rate with 1-AQ addition

**Fig. 4:**
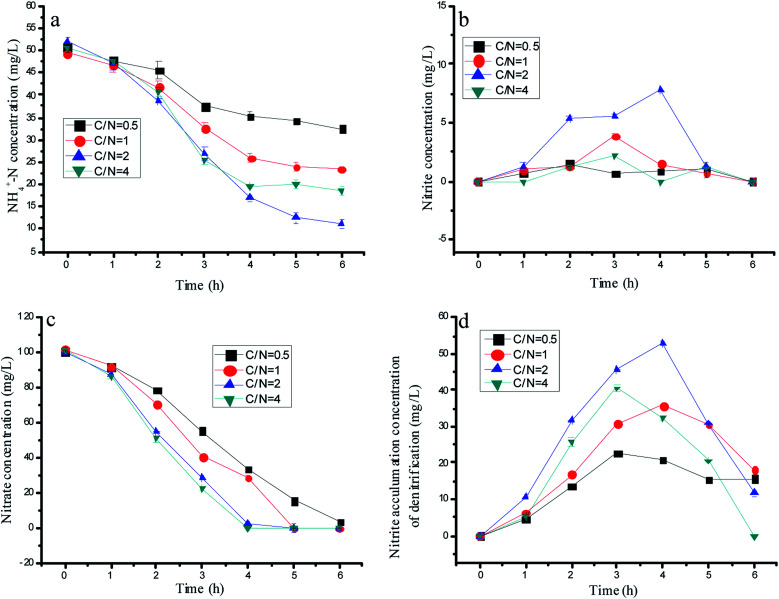
The variation of the nitrogen concentration with optimal 1-AQ addition at different C/N ratios in batch experiments.

Many reports in the literature proved that a suitable C/N is a key for achieving nitrite accumulation by partial denitrification. There would be nitrate residual if the carbon source was limited, and the accumulated nitrite could be reduced when the carbon source was abundant. Thus, two parallel experiments would be established to explore the effects of the C/N ratio on the nitrogen removal performance by mixed bacteria and a single denitrification biomass with 75 µM 1-AQ addition. As shown in Fig. 4d, a significant nitrite accumulation was observed in each test during denitrification. The maximum nitrite accumulation amount in the single denitrification experiments was 53.0 mg L^−1^ at C/N = 2 corresponding to 20.8, 35.8 and 32.2 mg L^−1^ at C/N = 0.5, 1 and 4, respectively. Due to the too slow nitrate conversion, the derisory carbon source (such as C/N = 0.5) also could bring about the deficient nitrite production. Based on the huge gap in the nitrite accumulation by denitrification with different C/N ratios, the ammonia removal performance showed the diverse tendency. For instance, when the C/N ratio was adjusted to 0.5, the ammonia removal efficiency was only 36.18%. However, ammonia could be removed much more quickly with the C/N ratio increased at a certain range. The topmost ammonia removal efficiency was 78.6% at the C/N ratio of 2.0, which were 2.17 and 1.49 fold higher than that of the C/N ratio of 0.5 and 1.0. Interestingly, the sufficient carbon source at C/N = 4 conversely reduced more nitrite by denitrification with the lower ammonia removal efficiency (Fig. 3a–c). Heterotrophic denitrification has been widely used for nitrogen removal from wastewater containing nitrate, but it needs external carbon sources and produces high amounts of waste sludge.^35,36^ In our study, nitrate only reduced to nitrite when the C/N ratio = 2, but a partial denitrification brought in less residual sludge compared with the complete denitrification.

### 3.5 Comparison of the nitrogen removal performance with different RMs on continuous experiments

**Fig. 5:**
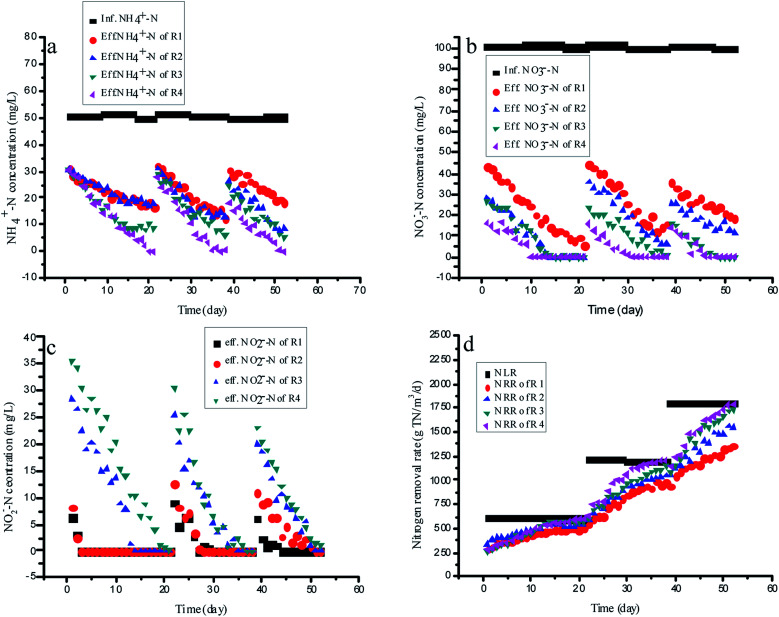
The variation of the nitrogen concentration with different RMs in continuous experiments.

Four identical upflow fixed-bed column reactors, R1 (the control reactor, without RM addition), R2 (with the ME addition of 75 mg L^−1^), R3 (with the AQ addition of 75 mg L^−1^) and R4 (with the 1-AQ addition of 75 mg L^−1^) were applied for continuous experiments with the external carbon source (glucose) as the organic carbon resource (C/N = 2). For enough nitrite production by partial denitrification, R1 without any RMs was still showing the same ammonia removal rate as other reactors by anammox during the initial startup period (Fig. 5A). Only three days later, the effluent nitrite of R1 and R2 were decreased to 0 quickly, although the nitrate removal efficiency of R2 with ME addition was 26.1% higher than that of the control (Fig. 5B and C). These results demonstrated that the ME addition could accelerate both nitrate and nitrite removal by denitrification biomass so that it was not suitable for mediating nitrite accumulation by partial denitrification. Unlike R1 and R2, the effluent nitrite of R3 and R4 with AQ and 1-AQ addition reached 28.2 and 35.7 mg L^−1^ in the initial startup period, which could ensure adequate nutrition for the anammox biomass in the continuous experiments. Gradually, the ammonia removal efficiency of R1 increased slowly from 39.4% to 66.6% due to a deficiency of nitrite within 21 days incubation. However, the ammonia removal efficiency of R3 and R4 by the anammox reaction showed a more pronounced upward tendency. On day 21, the ammonia removal rate of R4 with 1-AQ addition reached 198.0 g N m^−3^ d^−1^, corresponding to the ammonia removal efficiency of 100%, which was 50.1% higher than that of the control (132.0 g TN m^−3^ d^−1^). Apparently, the more excellent ammonia removal performance of anammox was relevant to RMs (AQ and 1-AQ) promoting the abundant nitrite accumulation by denitrification. Also, benefitting the distinguished anammox reaction, the TN removal rate of R3 (565.7 g TN m^−3^ d^−1^) and R4 (599·3 g TN m^−3^ d^−1^) was enhanced by 10.6% and 17.2% compared with R1 (511.6 g TN m^−3^ d^−1^).

Then, the nitrogen loading rates of all reactors were increased by shortening HRT with the constant influent substrate concentrations to investigate the long-term effects of RMs on the integrating process. In these experiments, the anammox reaction of the integrating process was the main research object, but not denitrification. Thus, HRT was adjusted on the basis of the effluent ammonia concentration. At the end of this experiment, the NRRs of R3 and R4 reached 1733.2 and 1788·4 g TN m^−3^ d^−1^, which was about 27.4 and 31.5% higher than that of R1 (1360.2 g TN m^−3^ d^−1^) when the NLRs of all reactors increased to 1801.2 g TN m^−3^ d^−1^ on day 188. The results of this study proved that nitrite was produced stably with a partial denitrification application with RM addition, which revealed an alternate efficient way to treat nitrogen wastewater by integrating with the autotrophic anammox process.

### 3.6 Biomass identification

Considering the different forms of two functional bacteria, 36 samples of dispersed sludge and 28 samples of embedding pellets taken from R4 were identified *via* 16S rDNA sequencing. The anammox biomass of *Candidatus Kuenenia* sp. was the primary group, which accounted for approximately 69.4% (25/36) of the total biomass in the dispersed sludge of the reactor, as illustrated in Table 1 and Fig. S3.† In addition, some kinds of denitrifying biomass accounted for about 16.7% of the whole biomass community, such as *Yersinia frederiksenii*, Flavobacteriaceae, *Trichococcus*, *Thauera* and *Klebsiella* genera. And, *Yersinia frederiksenii* and *Thauera* were regarded as partial denitrification, which have the capacity to reduce nitrate to nitrite, but not further to nitrogen gas.^15,37^ Also, *Yersinia frederiksenii* and *Thauera* were the dominant groups with a percentage of 32.1% and 14.3% in the embedding pellets by the phylogenetic classification according to Table 2 and Fig. S4,† which might be responsible for the high nitrite accumulation in this study. *Hydrogenophaga*, *Trichococcus*, *Klebsiella* and *Dechloromonas* genera were also considered denitrification biomass. It was determined that the denitrifying biomass accounted for about 75% of the whole biomass community. In addition, the anammox biomass of *Candidatus Kuenenia* sp. was the fraction group (2/28) after continuous incubation in the embedding pellets.

**Table 1:** BLAST analysis of 16S rDNA sequences in dispersed sludge of R4

No. of clones	Closest relative	Accession	Similarity (%)
25/36	*Candidatus Kuenenia* sp.	HM769655	100%
3/36	*Yersinia frederiksenii*	AJ639880	99–100%
1/36	*Flavobacteriaceae*	AM179864	100%
2/36	*Thauera*	AB021377	99%
2/36	*Klebsiella* genera	AJ233420	99%
2/36	*Trichococcus*	AB755786	99%
1/36	*Dyella* sp.	MF370623	99%

**Table 2:** BLAST analysis of 16S rDNA sequences in the embedding pellets of R4

No. of clones	Closest relative	Accession	Similarity (%)
9/28	*Yersinia frederiksenii*	AJ639880	99–100%
4/28	*Hydrogenophaga*	AB021420	100%
4/28	*Thauera*	AB681853	99%
2/28	*Candidatus Kuenenia* sp.	MK353155	100%
2/28	*Klebsiella* genera	AJ233420	99%
2/28	*Zoogloea*	AB201043	99%
1/28	*Trichococcus* sp.	MH569475	99%
1/28	Uncultured bacterium gene clone: *PSAE067*	AB533457	98%
1/28	*Sulfurospirillum halorespirans* strain *PCE-M2*	NR_028771	98%
1/28	*Cloacibacterium* sp.	MK770612	100%
1/28	*Dechloromonas*	AB769215	100%

Recently, different research directions of anammox bacteria were followed with interests by scientists, such as resuscitation after dormancy,^38^ identification of a new species and its characteristics,^39^ analysis of the cellular structure^40^ and the engineering application of sidestream and mainstream.^41^ Some researchers' work could bend to increase the nitrite productivity rate by ammonia-oxidizing bacteria.^42^ Researchers found that partial denitrification also could produce nitrite, but it was not constant and unstable.^37^ Also, Wang *et al.* reported that a partial denitrification could couple with the immobilization of anammox in a continuous upflow reactor with a nitrogen removal efficiency of 88.5%.^43^ Our research focused on accelerating the nitrite accumulation of denitrification by RM addition, which provided another way of thinking to integrate the anammox process for nitrogen removal. With 1-AQ addition, the nitrite accumulation increased 1.55-fold with C/N = 2 by denitrification compared with that of the control, and the NRRs of the integrating process reached 1801.2 g TN m^−3^ d^−1^ through 188 days incubation.

The mechanism that RMS could accelerate the bacteria activities was also controversial. Qiao *et al.* revealed that RMS could catalyze the enzyme activities instead of coenzyme Q.^28^ The addition of RMs might expedite the electron transfer between the electron acceptors and donors. Van der Zee *et al.* recognized that part of these differences may be directly related to the E0′ of the shuttling compounds investigated.^44^ In addition, other physico-chemical properties of the shuttling compound may play a role in determining its feasibility and strength as a mediator for azo-dye reduction. However, it was regretful that the method for how to choose the optimal RMs for the special biomass to treat contaminants did not have a unified view. In our study, the purpose of the RM addition was to increase the nitrite accumulation. Thus, the chosen RMs could accelerate the NR enzyme activities even higher than the NIR activities. And, the much bigger gaps of NR and NIR activities by partial denitrification had, the better we would want.

## 4. Conclusion

In this study, it was demonstrated that the nitrite accumulation *via* partial denitrification with 1-AQ could be increased 86.1% at the optimal dosing concentration of 75 µM. The NRR of the reactor *via* immobilizing partial denitrification biomass and 1-AQ to integrate with anammox process reached 1788.4 g TN m^−3^ d^−1^ using only ammonia and nitrate as the influent nitrogen resources in the long-term operation. The 16S rDNA sequencing results proved that the partial denitrification species was the primary group of the denitrifying biomass.

## Conflicts of interest

We declare that we have no financial and personal relationships with other people or organizations that can inappropriately influence our work, there is no professional or other personal interest of any nature or kind in any product, service and/or company that could be construed as influencing the position presented in, or the review of, the manuscript entitled.

## Supplementary Material

RA-009-C9RA05525H-s001
