# Supplementary material for: Immobilizing partial denitrification biomass and redox mediators to integrate with the anammox process for nitrogen removal
Source: RSC Adv. 2019 Dec 13;9(70):41351–60. doi: 10.1039/c9ra05525h (PMC9076434; doi:10.1039/c9ra05525h)
Supplement: RA-009-C9RA05525H-s001 [file RA-009-C9RA05525H-s001.pdf]

Electronic Supplementary Material (ESI) for RSC Advances.
This journal is © The Royal Society of Chemistry 2019

Co-immobilizing partial denitrification biomass and redox mediators to integrate with anammox process for nitrogen removal

Chuan He [1], Li'e Wei [1], Faying Lai [1], Chunhuo Zhou [1], Guorong Ni [1], Jianmin Hu [2], Xin Yin [1,2*]

1 Nanchang Key Laboratory of Nutrition Management of Crops , Prevention and Controlling of agricultural non-point source pollution，College of Land Resource and Environment, Jiangxi Agricultural University, Nanchang 330045, PR China

2 Jiangxi Provincial Key Laboratory of Water Resources and Environment of Poyang Lake, Jiangxi Institute of Water Sciences, Nanchang 330029, PR China

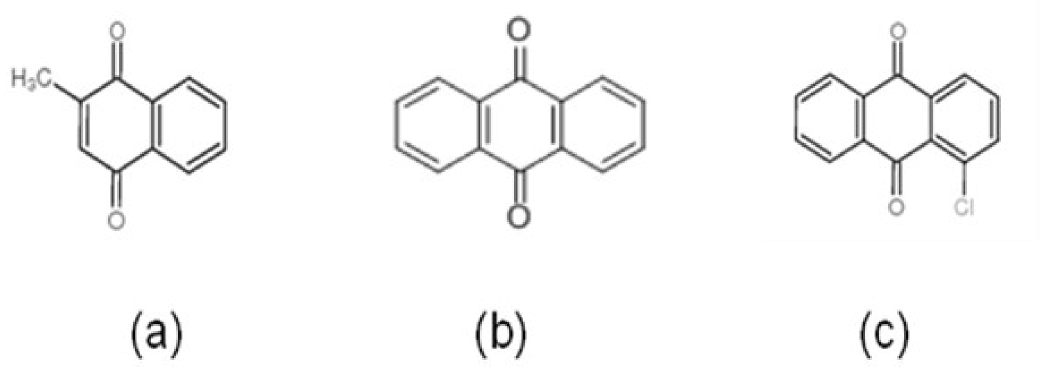

Fig. S1 the structure of the three RMs. (a) was ME, (b) was AQ, (c) was 1-AQ.

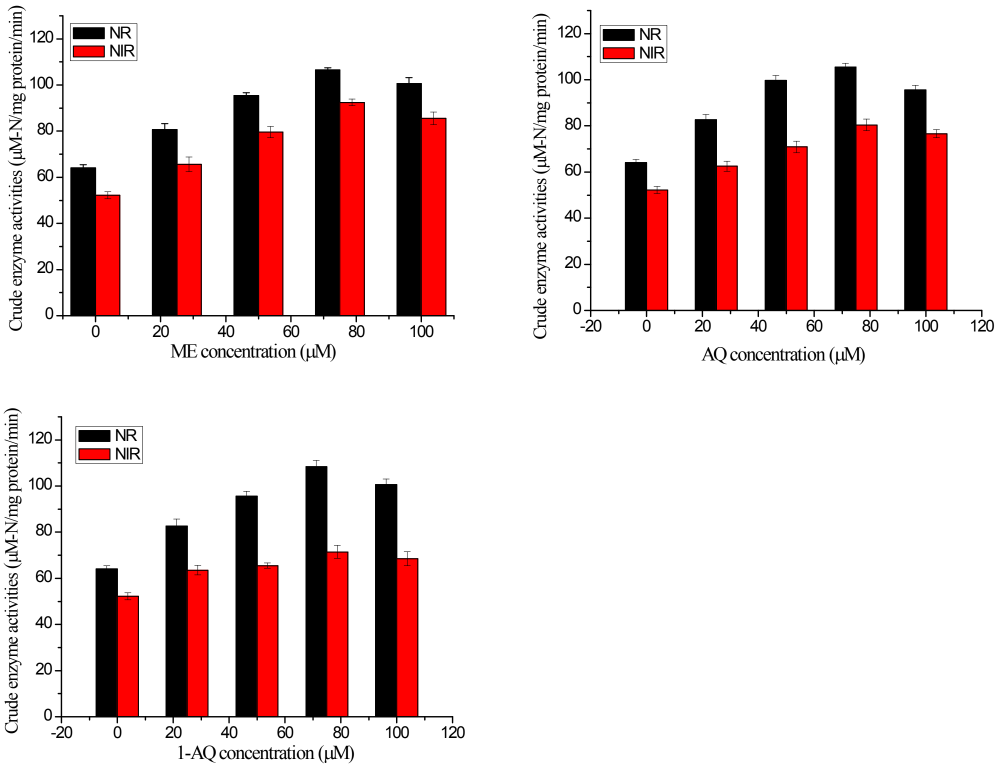

Fig.S2 Effects of RMs on the NR and NIR activities of denitrification biomass

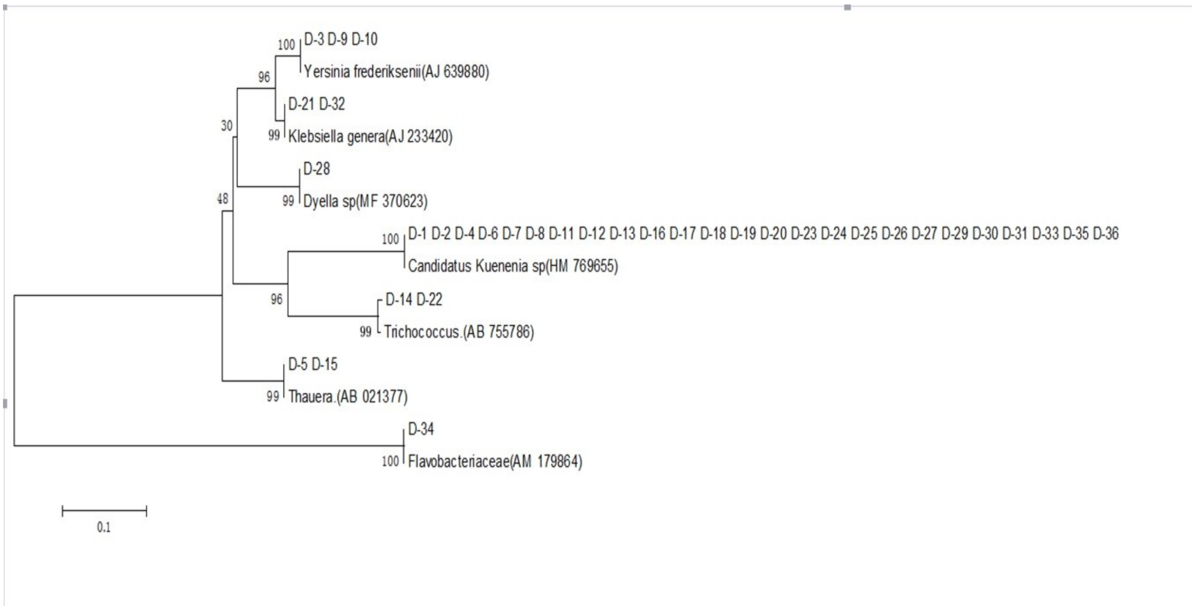

Fig. S3. Phylogenetic tree of samples taken from tdispersed sludge of R4

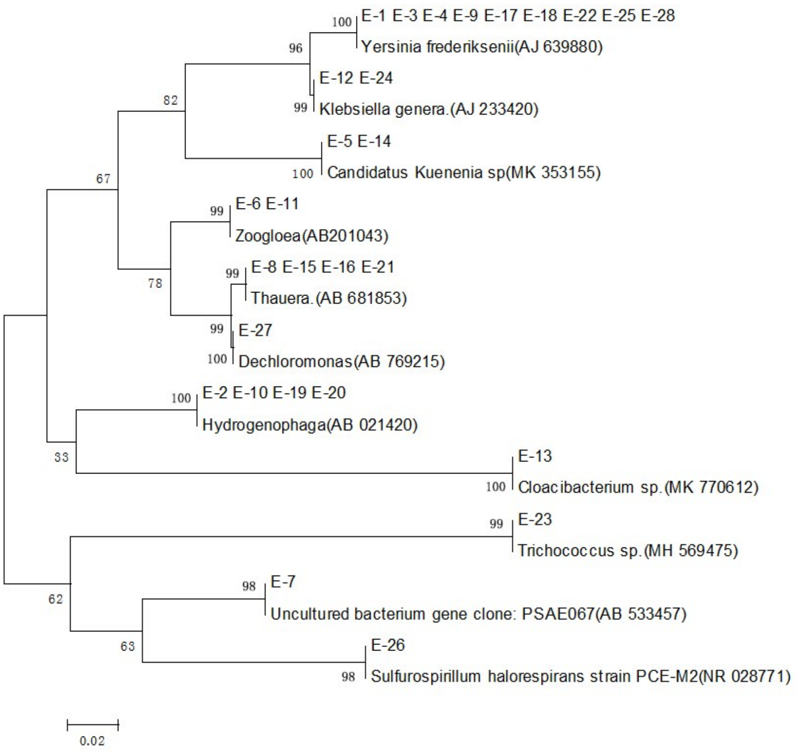

Fig. S4. Phylogenetic tree of samples taken from the embedding pellets of R4
